# Contextual and Individual Variables as Predictors of Energy-Dense Meals in Food Choices

**DOI:** 10.3389/fpsyg.2022.803326

**Published:** 2022-07-06

**Authors:** Daniela Borbón-Mendívil, Cesar Tapia-Fonllem, Blanca Fraijo-Sing

**Affiliations:** Programs of Master and Doctorate in Psychology, University of Sonora, Hermosillo, Mexico

**Keywords:** eating situation, contextual variables, food choice, individual variables, energy-dense meals

## Abstract

Diets based on meals that provide a large amount of energy and consumed frequently often increase the rate of growth of the body mass index (overweight or obesity) and, in turn, the risk of suffering from non-communicable diseases. In order to make a food choice, it is necessary to search for foods in the environment, taking into account physical and social variables (contextual variables) which, together with individual variables, delimit the situation of food selection. The objective of this study was to evaluate the effect of social facilitation, portion size, salience of food, and food preference or rejection on the selection of energy-dense foods by young college students. To do so, we performed a factorial experiment in which unaccompanied and accompanied participants (levels of social facilitation) as they went through the process of choosing from different options of main dishes, beverages, and desserts then noted the reasons for their selection (preference or rejection of the food). Results showed significant differences between the group of accompanied participants and salience of food in the selection of the energy-dense main dishes and desserts (pizza, spaghetti, and chocolate cake). A significant relationship was also identified between accompanied participants, hedonistic/sensory reasons (food preference or rejection category), and salience of food in the selection of the energy-dense main dishes. In conclusion, key findings of the variables that constitute the situation that predicts the selection of energy-dense foods have emerged from this study, when participants and the given level of social facilitation (in this case, being accompanied) were faced with the conditions of the food salience of the meals of their preference regarding its taste and appearance.

## Introduction

Diet refers to the voluntary and personal behavior to obtain, prepare, and ingest food. To obtain them, people must adapt to the environment and go through the process of food choice, which is understood as taking daily decisions about the food that involve discriminating and choosing meals that will be consumed in accordance with the time of day. Moreover, there are also choices regarding where to eat, with whom to eat, or where to buy meals. This process enables individuals to fraction their daily portions of food both daily and during the day, based on their habits and circumstances by determining the consumption and frequency of meals. Therefore, the environment is the means for the organism to acquire necessary nutrients (Cervera et al., 2004; Poelman and Steenhuis, 2019; Martín and Cantarero, 2020). More specifically, a diet that provides a large number of kilocalories that are not used as energy increases the accumulation of fat in the body, classified as overweight and obesity, which increases the risk of suffering non-communicable diseases and accounts for 71% of deaths worldwide in people between 30 and 69 years of age).

This topic has been studied using the concept of reference event, i.e., any event of interest related to food, e.g., menu planning or analysis of the bite of a food. The reference event is analyzed per unit of reference, and it could be a meal, a dish, or an eating pattern. In this study, the reference event is food selection and reference units are understood as meal formats, i.e., the size of a meal involving the combination of solid and liquid elements (Rozin and Tourila, 1993; Bell and Meiselman, 1995; Meiselman, 1996, 2006; Mäkelä, 2000; Mäkelä and Nivi, 2019).

The food choice process involves individual variables, e.g., expectations, habits, experiences, sensory, physiological, behavioral responses, and food characteristics such as odor, appearance, and taste. In accordance with these variables, people also interact with the variables of the context, i.e., the events and physical and social factors of the environment that influence the perception of the event of reference and, thus, the situation under which the food choice is made is formed (Rozin and Tourila, 1993; Bell and Meiselman, 1995; Meiselman, 1996, 2006).

In this study, individual variables have been analyzed using the concept of food preference or rejection proposed by Rozin (2007) whose categories of this concept were further developed based on the study by Rozin and Fallon (1980) and are defined as follows:

(1) *Hedonistic or sensorial reasons:* Preference or rejection determined by the taste, texture, smell, or appearance of food. The indicators in this category are understood as properties that a person evaluates subjectively (intrinsic factors) and the evaluation, in turn, is based on cultural or religious factors from a region, and on environmental factors (extrinsic factors), e.g., situational, marketing, and time variables (Khan, 1981; Shepherd and Sparks, 1994).

(2) *Anticipated consequences:* They could be related to beliefs about whether a food is dangerous, or beneficial to health, and also whether that food carries relatively rapid positive or negative post-ingestive consequences that can influence health. For example, a person might reject a food because he or she is allergic to it, or because he or she considers it to be high in energy (Rozin, 2007).

Contextual variables are understood as characteristics that are physically present during the food choice process, but that are not the food itself, e.g., labeling, packaging, the place where the food is presented (i.e., at a restaurant or at home), and additional to the presence of other people (Rozin and Tourila, 1993; Bell and Meiselman, 1995; Wansink and Sobal, 2007). In particular, Wansink and Sobal (2007) and Wansink (2004) understand these variables as environmental influences and organize them into two dimensions:

(1) *Food environment*: It refers to the presentation of food, e.g., portion size, as to say, a consumption norm that involves elements with which people normally interact on a daily basis and are indicators of how much to eat and when to finish eating (Wansink and Sobal, 2007) and Salience of Food or preponderance which refers to the storage of food products prominently or preponderantly at the point of consumption because they may take up more storage space, they can sometimes be packaged in unusual ways (promotional packages) and can be placed in visible locations, e.g., on the counter in front of the pantry (Chandon and Wansink, 2002).

(2) *Eating environment*: This involves social interactions in the dietary situation, including the concept of social facilitation, i.e., the presence of more people during a segment of the diet, whose company affects the amount of food to be consumed (Stroebele and De Castro, 2004).

In accordance with the above, there is evidence of the influence of some contextual variables, specifically the ones related to environmental properties. For example, in a study designed on a digital environment, salience was manipulated by varying brightness levels of meals regarded as healthy and non-healthy. The influence of salience of healthy products on food choice was also tested, even if the non-healthy product was regarded as better-tasting. Considering this, there is a belief that this factor is relevant when choosing food; even so, it is necessary for its measurement to be performed with a basis on complex decision-making, such as a meal’s real eating environment (Dai et al., 2020).

Food portion size is considered to predict beverage choice; however, in a study performed by Ferrar et al. (2019), it was proven that given a variety of portion sizes in main dishes, beverage choice was taken according to the subjects’ familiarity with the product (water or soft drink); yet the probability of choosing the better-known beverage increased if a larger portion was chosen.

An eating situation involves social interactions, which means that being in the presence of other people while having a meal has an impact on the amount of food consumed. This is known as social facilitation (Stroebele and De Castro, 2004). If the number of people influences the amount consumed, then it possibly also influences food choice (Bell and Meiselman, 1995) since it is believed that meal consumption is a matter of choice (Kjaernes and Holm, 2007).

Classically, food choice has been studied using images for its measurement and focused on the analysis of the selection of main dishes, snacks, or beverages, separately. However, the eating event involves more than one food and one beverage (Oltersdorf et al., 1999; Meiselman, 2008). Therefore, in this study, the measurement of variables was designed with real foods, which formed the plate or structured plates containing more than one food that are consumed at any time of the day (Meiselman, 1996, 2000). The objective of the study was to evaluate the effect of social facilitation, portion size, salience of food (contextual variables), and food preference or rejection (individual variables) on the selection of energy-dense meals (Meiselman, 1996, 2000).

## Methodology

### Participants

A total of 22 students aged 20–24 years (*M* = 21.8, σ = 0.958) from the sixth and eighth semesters of the medicine and clinical biochemist majors in the University of Sonora, Cajeme campus, Mexico, were participated. Due to the demand of this study and the time and space availability in the university, participant selection was performed *via* the non-probabilistic technique of convenience type. This means that a sample was selected from an easy-to-access population, which prevents knowing the probability of participants being chosen (Ochoa, 2015).

### Preparing and Presenting the Meals

The meal was composed of a main dish, dessert, and beverage. Everything was prepared in a homemade fashion, with known and frequently consumed foods by the participants. Tables 1, 2 show the ingredients and amounts present in the portions and their kilocalorie content. Participants who belonged to groups entered a room where there were several tables together, two types of dishes, desserts, and beverages available to each one of them.

**TABLE 1:** Chi-square results of main course, beverage, and dessert selection in session 1.

Conditions for choosing according to portion size	Social facilitation levels	Values of X^2^
Main dish	Group 1: Unaccompanied Participants Group 2: Accompanied Participants	*X^2^* = 1.575 (*df* = 2), *p* > 0.05. *X^2^* = 0.917 *(df* = 1), *p* > 0.05.
Beverage	Group 1: Unaccompanied Participants Group 2: Accompanied Participants	*X^2^* = 1.479, (*df* = 3), *p* > 0.05. *X^2^* = 3.600 (*df* = 4), *p* > 0.05.
Dessert	Group 1: Unaccompanied Participants Group 2: Accompanied Participants	*X^2^* = 0.321 (*df* = 1), *p* > 0.05. *X^2^* = 0.020, (*df* = 1), *p* > 0.05.

**TABLE 2:** Chi-square results of main course, beverage, and dessert selection in session 2.

Conditions for choosing according to portion size	Social facilitation levels	Values of X^2^ per Group
Main Dish	Group 1: Unaccompanied Participants Group 2: Accompanied Participants	*X^2^* = 1.397 (*df* = 2), *p* > 0.05. *X^2^* = 3.208 (*df* = 4), *p* > 0.005.
Beverage	Group 1: Unaccompanied Participants Group 2: Accompanied Participants	*X^2^* = 3.606 (*df* = 3), *p* > 0.05. *X^2^* = 5.238, (*df* 2), *p* > 0.05.
Dessert	Group 1: Unaccompanied Participants Group 2: Accompanied Participants	*X^2^* = 3.654 (*df* = 3), *p* > 0.05. *X^2^* = 7.222 (*df* = 2), *p* < 0.05. (Contingency coefficient = 0.64, *p* < 0.05.)

### Devices and Material

The sessions took place in a Gesell chamber at the laboratory of the University of Sonora which is equipped with computer devices that control three cameras installed in the room where the behavioral analysis occurs. There were four tables where all the food products were presented in biodegradable, disposable materials along with napkins and silverware. The meals were served at room temperature; however, participants had at their disposal a microwave within the facilities to heat their meals if they wished to do so. Beverages were presented at a cold temperature; therefore, they were kept inside an icebox filled with ice to maintain a good temperature. There were also white sheets of paper, pens, pencils, and other stationery items to record both the assistance and the motives for which the products were chosen by the participants in each session.

### Procedure

The experiment was conducted on February 2020. Through the informed consent letter, all participants were made aware of the objective of the study and the activities to be performed and were asked for authorization to be filmed. The participants were separated into two groups and four sessions were assigned to each group. According to the group they belonged to, each participant entered the Gesell chamber of the University of Sonora where there were several tables, and two types of main dishes, two types of beverages, and two types of desserts available to be chosen for consumption were presented on the tables. Fifteen minutes were allotted for the activity, and when they concluded the task, they wrote down the reasons for selecting their food in a sheet of paper.

### Data Analysis

To identify differences between groups or levels of social facilitation and portion size and food preponderance conditions, main effects were observed and pairwise comparisons were performed using analysis of variance (ANOVA) with repeated measures (RMs) and *post-hoc* tests with Bonferroni adjustment. To identify the degree of relationship between variables, three-dimensional contingency tables were constructed with the chi-square significance test (X^2^) as a measure of association.

## Results

### Interaction Effects Between Social Facilitation Levels and Portion Size

Regarding the interactions between social facilitation levels and portion size levels, no significant differences were found (*F* = 1.016, *df* = 5.0, 15.0, *p* > 0.05, η^2^ = 0.26).

### Interaction Effects Between Levels of Social Facilitation and Salience of Food

The interactions between the levels of social facilitation and the levels of food preponderance indicated that there are significant differences between the levels of social facilitation and the levels of food salience (*F* = 4.31, *df* = 5.0, 14.0, *p* < 0.05, η^2^ = 0.60). According to the Bonferroni *post-hoc* test, such significant differences are found in the selection of dessert in session 3 depending on whether the participants were unaccompanied (*M* = 1.10, σ = 0.316, *p* < 0.05) or accompanied (*M* = 1.60, σ = 0.516). Significant differences were also found in the selection of the main dish in session 4 (*M* = 1.50, σ = 0.527, *p* = 0.05), while also when participants were accompanied in this session (*M* = 1.10, σ = 0.316).

### Degree of Association Between Portion Size, Social Facilitation, and Food Preference or Rejection

According to Tables 1, 2, the values indicate that there are no significant associations between levels of social facilitation, energy-dense main course, beverage and dessert selections according to portion size, and food preference or rejection categories. Therefore, neither hedonistic/sensory reasons nor anticipated consequences or portion size are related to the selections participants made.

### Degree of Association Between Salience of Food, Social Facilitation, and Food Preference or Rejection

The values in Table 3 indicate that there are no associations in levels of social facilitation, the preponderance of foods in their conditions, and food preference or food rejection categories. Therefore, it is assumed that the choices participants made were not related to hedonistic/sensory reasons or anticipated consequences.

**TABLE 3:** Chi-square results of main course, beverage, and dessert selection in session 3.

Conditions for choosing according to portion size	Social facilitation levels	Values of X^2^
Main Dish	Group 1: Unaccompanied Participants Group 2: Accompanied Participants	*X^2^* = 0.278, (*df* = 1), *p* > 0.05. *X^2^* = 1.270, (*df* = 1), *p* > 0.05.
Beverage	Group 1: Unaccompanied Participants Group 2: Accompanied Participants	*X^2^* = 0.900 (*df* = 2), *p* > 0.05. *X^2^* = 4.518 (*df* = 2), *p* > 0.05.
Dessert	Group 1: Unaccompanied Participants Group 2: Accompanied Participants	*X^2^* = 0.278 (*df* = 2), *p* > 0.05. *X^2^* = 2.037 (*df* = 2), *p* > 0.05.

Table 4 shows that there was only a statistically significant association between accompanied participants, main course selection (pizza and spaghetti) in preponderance condition, and hedonistic/sensory reasons.

**TABLE 4:** Chi-square results of main course, beverage, and dessert selection in session 4.

Conditions for choosing according to portion size	Social facilitation levels	Values of X^2^ per group
Main Dish	Group 1: Unaccompanied Participants Group 2: Accompanied Participants	*X^2^* = 2.500 (*df* = 1), *p* > 0.005. *X^2^* = 9.000 (*df* = 2), *p* < 0.05. (Contingency coefficient = 0.70, *p* < 0.05).
Beverage	Group 1: Unaccompanied Participants Group 2: Accompanied Participants	*X^2^* = 2.744 (*df* = 1), *p* > 0.05. *X^2^* = 1.667(*df* = 1), *p* > 0.05.
Dessert	Group 1: Unaccompanied Participants Group 2: Accompanied Participants	No statistics were computed for this group because the observed and expected frequencies have the same values. *X^2^* = 0.625 (*df* = 1), *p* > 0.05.

## Discussion

This study confirmed the influence of contextual variables in the selection of energy-dense foods. Specifically, for accompanied participants, facing condition of the salience of foods with higher energy density, it was mainly the participant’s context which determined the selection of the main course (pizza and spaghetti) and dessert (a piece of chocolate cake) with said properties. A significant relationship was also found between context and hedonistic or sensory reasons (individual component). These variables shaped the situation of energy-dense food selection. These results show that eating behavior is mainly related to the elements of the environment where individuals behave, i.e., food choices are situational or circumstantial (Doucerain and Fellows, 2012; Sobal et al., 2014).

The social factor is considered to be one of the main components affecting food choice (Birkenhead and Slater, 2015), even though there are few experimental studies that examine social facilitation in relation to food selection (Ruddock et al., 2019). Thus, this study demonstrates the relevance of the environment on eating behavior, specifically on food choice. The results encourage considering other variables for future testing, e.g., cultural norms or group processes, considered social affordances, are risky contextual features for increased body fat and cardiovascular disease. Making the choice to buy or not to buy and to consume a food or not implies a social and physical posture of the environment which enables dietary patterns (Carrus et al., 2018).

## Data Availability Statement

The datasets analyzed for this study can be obtained by contacting the correspondent author, these will be made available by the authors, without undue reservation.

## Ethics Statement

The studies involving human participants were reviewed and approved by the Comité de Ética en Investigación de la Universidad de Sonora. Written informed consent to participate in this study was provided by the participants’ legal guardian/next of kin. The patients/participants provided their written informed consent to participate in this study. The individual(s) provided their written informed consent for the publication of any identifiable images or data presented in this article.

## Author Contributions

DB-M contributed to the conceptualization, design of this study, and acquisition of data, ran formal analysis, and organized databases. CT-F contributed by supervising this study, its methodological tasks, and data interpretation. BF-S made substantial contributions by editing and revising the manuscript critically for important intellectual content. DB-M and CT-F provided the writing of the original draft. All authors contributed to the manuscript revision and read and approved the submitted version.

## Conflict of Interest

The authors declare that the research was conducted in the absence of any commercial or financial relationships that could be construed as a potential conflict of interest.

## Publisher’s Note

All claims expressed in this article are solely those of the authors and do not necessarily represent those of their affiliated organizations, or those of the publisher, the editors and the reviewers. Any product that may be evaluated in this article, or claim that may be made by its manufacturer, is not guaranteed or endorsed by the publisher.
