# Supplementary material for: Contextual and Individual Variables as Predictors of Energy-Dense Meals in Food Choices
Source: Front Psychol. 2022 Jul 6;13:803326. doi: 10.3389/fpsyg.2022.803326 (PMC9298269; doi:10.3389/fpsyg.2022.803326)
Supplement: Supplementary file 1 [file Data_Sheet_1.PDF]

frontiers

Supplementary Material

Menu	Portion in grams or milliliters		Total kilocalories	
	Small portion	Large portion	Small portion	Large portion
Main dish				
Chicken breast cooked with vegetables	100 g	150 g	354 kcal	458 kcal
Cooked white rice	60 g	60 g		
Steamed vegetables (broccoli, cauliflower, and carrot)	100 g	100 g		
Pork chorizo cooked with white potatoes	100 g	140 g	447 kcal	708 kcal
Corn tortillas	60 g	90 g		
Refried beans	50 g	50 g		
Dessert				
Mandarin and pineapple	64	124	34 kcal	62 kcal
Cookies	45.6 g (4 pieces)	114 g (10 pieces)	216 kcal	540 kcal
Beverages				
Cola soft drink	235 ml	600 ml	71 kcal	180 kcal
Natural water	335 ml	500 ml	0	0

Supplementary Table 1. Main dishes, desserts, and beverages for portion size condition.

Salience in Product Storage		
Menu	Portion in grams or milliliters	Total Kilocalories
Main dish		
Beef steak cooked with chili, tomato, and onions	100 g	
Steamed vegetables (broccoli, cauliflower, and carrot)	100 g	405 kcal
Corn tortilla	60 g	
Pizza slices	100 g	
Pasta with tomato sauce	195 g	540
Beverages		
Cola soft drink	235 ml	71 kcal
Natural water	335 ml	0 kcal
Dessert		
Chocolate cake	28 g	104 kcal
Carrot cake	28 g	112 kcal

Supplementary Table 2. Main dishes, desserts, and beverages for salience of food condition.

		Dimensions of food preference or rejection		
Social facilitation levels	Beverage choice	Sensory/ hedonic reasons	Anticipated consequences	Total
Group 1: Unaccompanied Participants	Cola soft drink 600 ml (180 kcal)	1	1	2
		.9	1.1	2.0
	Cola soft drink 235ml (71 kcal)	2	1	3
		1.4	1.6	3.0
	Natural water 500 ml (0 kcal)	2	1	3
		1.4	1.6	3.0
	Natural water 335 ml (0 kcal)	0	3	3
		1.4	1.6	3.0
	Total	5	6	11
		5.0	6.0	11.0
Group 2: Participants accompanied by a classmate	Cola soft drink 235ml (71 kcal)	0	3	3
		1.1	1.9	3.0
	Natural water 500 ml (0 kcal)	2	0	2
		.7	1.3	2.0
	Natural water 335 ml (0 kcal)	2	4	6
		2.2	3.8	6.0
	Total	4	7	11
		4.0	7.0	11.0
Total	Cola soft drink 600 ml (180 kcal)	1	1	2
		.8	1.2	2.0
	Cola soft drink 235ml (71 kcal)	2	4	6
		2.5	3.5	6.0
	Natural water 500 ml (0 kcal)	4	1	5
		2.0	3.0	5.0
	Natural water 335 ml (0 kcal)	2	7	9
		3.7	5.3	9.0
	Total	9	13	22
		9.0	13.0	22.0

Supplementary Table 3. Food preference or rejection related to beverage choice (session 2).

		Dimensions of food preference or rejection		
Social facilitation levels	Dessert choice	Sensory/hedonic reasons	Anticipated consequences	Total
Group 1: Unaccompanied Participants	Tangerine and pineapple 64 gr (34 kcal)	1	1	2
		.7	1.3	2.0
	Tangerine and pineapple 124 gr (62 kcal)	3	2	5
		1.8	3.2	5.0
	Cookies 45.6 gr (4 units, 216 kcal)	0	2	2
		.7	1.3	2.0
	Cookies 114 gr (10 units, 540 kcal)	0	2	2
		.7	1.3	2.0
	Total	4	7	11
		4.0	7.0	11.0
Group 2: Participants accompanied by a classmate	Tangerine and pineapple 64 gr (34 kcal)	0	2	2
		1.2	.8	2.0
	Tangerine and pineapple 124 gr (62 kcal)	5	0	5
		3.0	2.0	5.0
	Cookies 114 gr (10 units, 540 kcal)	1	2	3
		1.8	1.2	3.0
	Total	6	4	10
		6.0	4.0	10.0

Supplementary Table 4. Preference or rejection of foods related to dessert choice (session 2).

		Dimension of food preference o rejection		
Social facilitation levels	Main dish choice	Sensory/hedonic reasons	Anticipated consequences	Total
Group 1: Unaccompanied Participants	Pizza and Spaghetti in a 295 gr portion (540 kcal)	4	2	6
		3.6	2.4	6.0
	Mexican Style Steak Stew in a 260 gr portion (405 kcal)	2	2	4
		2.4	1.6	4.0
	Total	6	4	10
		6.0	4.0	10.0
Group 2: Participants accompanied by a classmate	Pizza and Spaghetti in 295 gr portion (540 kcal)	5	1	6
		4.2	1.8	6.0
	Mexican Style Steak Stew in a 260 gr portion (405 kcal)	2	2	4
		2.8	1.2	4.0
	Total	7	3	10
		7.0	3.0	10.0
Total	Pizza and Spaguetti in 295 gr portion (540 kcal)	9	3	12
		7.8	4.2	12.0
	Mexican Style Steak Stew in a 260 gr portion (405 kcal)	4	4	8
		5.2	2.8	8.0
	Total	13	7	20
		13.0	7.0	20.0

Supplementary Table 5. Food preference or rejection related to main dish choice (Session 3).

Social facilitation levels	Beverage choice	Dimensions of food preference or rejection			
		State of environment	Sensory/hedonic reasons	Anticipated consequences	Total
Group 1: Unaccompanied Participants	Cola soft drink 235ml (71 kcal)	1	2	2	5
		.6	2.2	2.2	5.0
	Natural water 335 ml (0 kcal)	0	2	2	4
		.4	1.8	1.8	4.0
	Total	1	4	4	9
		1.0	4.0	4.0	9.0
Group 2: Participants accompanied by a classmate	Cola soft drink 235ml (71 kcal)	1	0	3	4
		.4	1.5	2.2	4.0
	Natural water 335 ml (0 kcal)	0	4	3	7
		.6	2.5	3.8	7.0
	Total	1	4	6	11
		1.0	4.0	6.0	11.0
Total	Cola soft drink 235ml (71 kcal)	2	2	5	9
		.9	3.6	4.5	9.0
	Natural water 335 ml (0 kcal)	0	6	5	11
		1.1	4.4	5.5	11.0
	Total	2	8	10	20
		2.0	8.0	10.0	20.0

Supplementary Table 6. Food preference or rejection related to beverage choice (session 3).

		Dimensions of food preference or rejection			
Social facilitation levels	Dessert choice	State of environment	Sensory/ hedonic reasons	Anticipated consequences	Total
Group 1: Unaccompanied Participants	Carrot cake 28 gr (104 kcal)	1	7	1	9
		.9	7.2	.9	9.0
	Chocolate cake 28 gr (112 kcal)	0	1	0	1
		.1	.8	.1	1.0
	Total	1	8	1	10
		1.0	8.0	1.0	10.0
Group 2: Participants accompanied by a classmate	Carrot cake 28 gr (104 kcal)	1	4	0	5
		.5	4.1	.5	5.0
	Chocolate cake 28 gr (112 kcal)	0	5	1	6
		.5	4.9	.5	6.0
	Total	1	9	1	11
		1.0	9.0	1.0	11.0
Total	Carrot cake 28 gr (104 kcal)	2	11	1	14
		1.3	11.3	1.3	14.0
	Chocolate cake 28 gr (112 kcal)	0	6	1	7
		.7	5.7	.7	7.0
	Total	2	17	2	21
		2.0	17.0	2.0	21.0

Supplementary Table 7. Preference or rejection of foods in relation to dessert choice (session 3).

Social facilitation levels	Main dish choice	Dimensions of food preference or rejection: State of environment	Sensory/ hedonic reasons	Anticipated consequences	Total
Group 1: Unaccompanied Participants	Pizza slices and pasta with tomato sauce 295 gr (540 kcal)		5	0	5
			4.0	1.0	5.0
	Beef steak 260 gr (405 kcal)		3	2	5
			4.0	1.0	5.0
	Total		8	2	10
			8.0	2.0	10.0
Group 2: Participants accompanied by one classmate	Pizza slices and pasta with tomato sauce 295 gr (540 kcal)	1	7	0	8
		.9	6.2	.9	8.0
	Beef steak 260 gr (405 kcal)	0	0	1	1
		.1	.8	.1	1.0
	Total	1	7	1	9
		1.0	7.0	1.0	9.0
Total	Pizza slices and pasta with tomato sauce 295 gr (540 kcal)	1	12	0	13
		.7	10.3	2.1	13.0
	Beef steak 260 gr (405 kcal)	0	3	3	6
		.3	4.7	.9	6.0
	Total	1	15	3	19
		1.0	15.0	3.0	19.0

Supplementary Table 8. Preference or rejection of foods in relation to the main dish choice (session 4).

		Dimensions of food preference or rejection		
Social facilitation levels	Beverage choice	Sensory/ hedonic reasons	Anticipated consequences	Total
Group 1: Unaccompanied Participants	Cola soft drink de 235ml (71 kcal)	2	1	3
		.9	2.1	3.0
	Natural water 335 ml (0 kcal)	1	6	7
		2.1	4.9	7.0
	Total	3	7	10
		3.0	7.0	10.0
Group 2: Participants accompanied by a classmate	Cola soft drink 235ml (71 kcal)	3	2	5
		2.0	3.0	5.0
	Natural water 335 ml (0 kcal)	1	4	5
		2.0	3.0	5.0
	Total	4	6	10
		4.0	6.0	10.0
Total	Cola soft drink 235ml (71 kcal)	5	3	8
		2.8	5.2	8.0
	Natural water 335 ml (0 kcal)	2	10	12
		4.2	7.8	12.0
	Total	7	13	20
		7.0	13.0	20.0

Supplementary Table 9. Food preference or rejection in relation to beverage choice (session 4).

		Dimensions of food preference or rejection		
Social facilitation levels	Dessert choice	Sensory/ hedonic reasons	Anticipated consequences	Total
Group 1: Unaccompanied Participants	Carrot cake 28 gr (104 kcal)	9	1	10
		9.0	1.0	10.0
	Total	9	1	10
		9.0	1.0	10.0
Group 2: Participants accompanied by one classmate	Carrot cake 28 gr (104 kcal)	6	2	8
		6.4	1.6	8.0
	Chocolate cake 28 gr (112 kcal)	2	0	2
		1.6	.4	2.0
	Total	8	2	10
		8.0	2.0	10.0
Total	Carrot cake 28 gr (104 kcal)	15	3	18
		15.3	2.7	18.0
	Chocolate cake 28 gr (112 kcal)	2	0	2
		1.7	.3	2.0
	Total	17	3	20
		17.0	3.0	20.0

Supplementary Table 10. Preference or rejection of foods in relation to dessert choice (session 4).

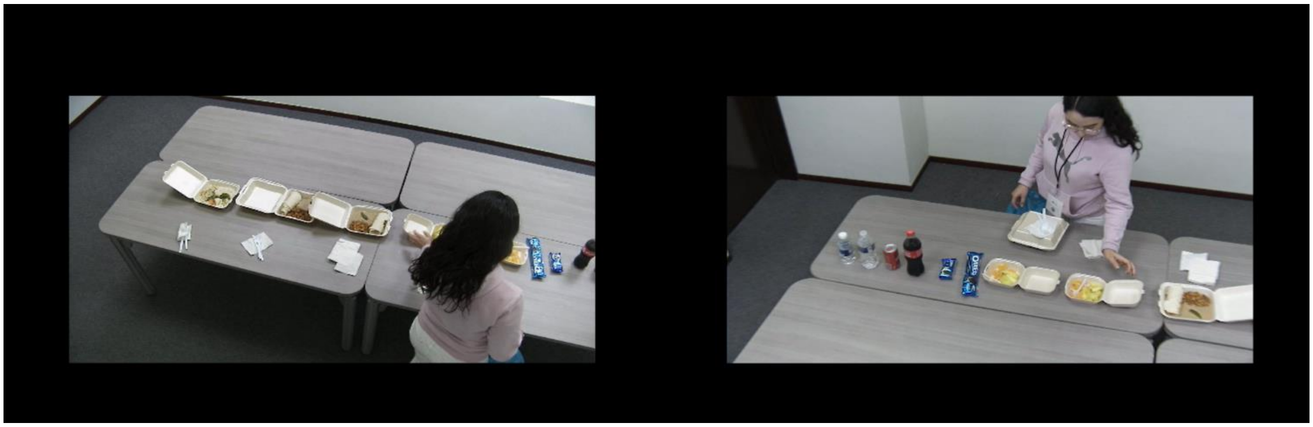

Supplementary Figure 1. Example of a preconditioned session to test the levels of the portion size factor with a participant from group 1 (social facilitation level).

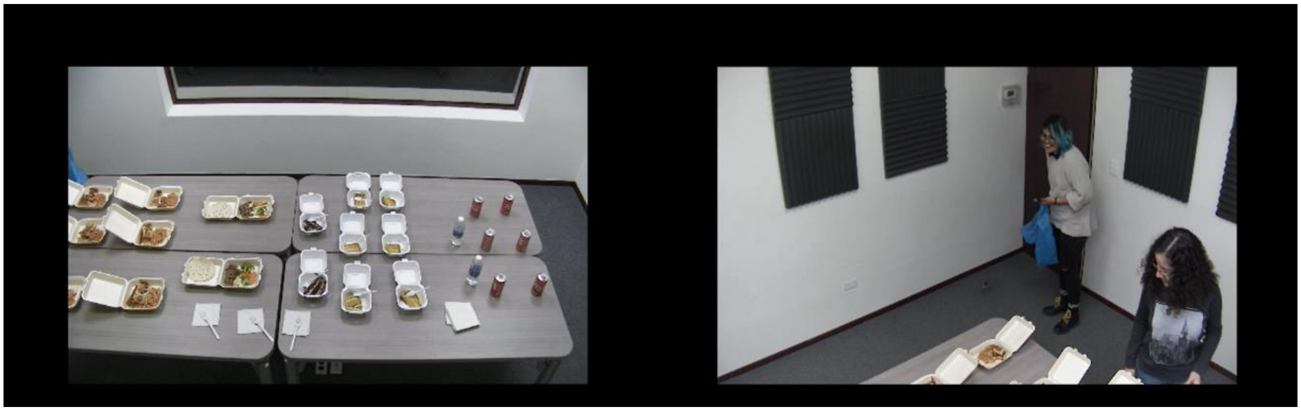

Supplementary Figure 2. Example of a preconditioned session to test the levels of the Salience of food factor with participants in group 2 (social facilitation level).

		Food Portion			Salience		
		Main Dish	Beverage	Dessert	Main Dish	Beverage	Dessert
Social Facilitation	Unaccompanied Participants	2 dish options with 2 portion sizes each	2 dish options with 2 portion sizes each	2 dish options with 2 portion sizes each	2 dish options	2 beverage options	2 dessert options
	Participants accompanied by one classmate	2 dish options with 2 portion sizes each	2 dish options with 2 portion sizes each	2 dish options with 2 portion sizes each	2 dish options	2 beverage options	2 dessert options

Supplementary Figure 3. Experimental Design 2x3x3.
